# Automated annotation of scientific texts for ML-based keyphrase extraction and validation

**DOI:** 10.1093/database/baae093

**Published:** 2024-09-27

**Authors:** Oluwamayowa O Amusat, Harshad Hegde, Christopher J Mungall, Anna Giannakou, Neil P Byers, Dan Gunter, Kjiersten Fagnan, Lavanya Ramakrishnan

**Affiliations:** Scientific Data Division, Lawrence Berkeley National Laboratory, 1 Cyclotron road, Berkeley, CA 94720, United States; Division of Environmental Genomics and Systems Biology, Lawrence Berkeley National Laboratory, 1 Cyclotron road, Berkeley, CA 94720, United States; Division of Environmental Genomics and Systems Biology, Lawrence Berkeley National Laboratory, 1 Cyclotron road, Berkeley, CA 94720, United States; Scientific Data Division, Lawrence Berkeley National Laboratory, 1 Cyclotron road, Berkeley, CA 94720, United States; DOE Joint Genome Institute, Lawrence Berkeley National Laboratory, 1 Cyclotron road, Berkeley, CA 94720, United States; Scientific Data Division, Lawrence Berkeley National Laboratory, 1 Cyclotron road, Berkeley, CA 94720, United States; DOE Joint Genome Institute, Lawrence Berkeley National Laboratory, 1 Cyclotron road, Berkeley, CA 94720, United States; Scientific Data Division, Lawrence Berkeley National Laboratory, 1 Cyclotron road, Berkeley, CA 94720, United States

## Abstract

Advanced omics technologies and facilities generate a wealth of valuable data daily; however, the data often lack the essential metadata required for researchers to find, curate, and search them effectively. The lack of metadata poses a significant challenge in the utilization of these data sets. Machine learning (ML)–based metadata extraction techniques have emerged as a potentially viable approach to automatically annotating scientific data sets with the metadata necessary for enabling effective search. Text labeling, usually performed manually, plays a crucial role in validating machine-extracted metadata. However, manual labeling is time-consuming and not always feasible; thus, there is a need to develop automated text labeling techniques in order to accelerate the process of scientific innovation. This need is particularly urgent in fields such as environmental genomics and microbiome science, which have historically received less attention in terms of metadata curation and creation of gold-standard text mining data sets. In this paper, we present two novel automated text labeling approaches for the validation of ML-generated metadata for unlabeled texts, with specific applications in environmental genomics. Our techniques show the potential of two new ways to leverage existing information that is only available for select documents within a corpus to validate ML models, which can then be used to describe the remaining documents in the corpus. The first technique exploits relationships between different types of data sources related to the same research study, such as publications and proposals. The second technique takes advantage of domain-specific controlled vocabularies or ontologies. In this paper, we detail applying these approaches in the context of environmental genomics research for ML-generated metadata validation. Our results show that the proposed label assignment approaches can generate both generic and highly specific text labels for the unlabeled texts, with up to 44% of the labels matching with those suggested by a ML keyword extraction algorithm.

## Introduction

**Figure 1.**
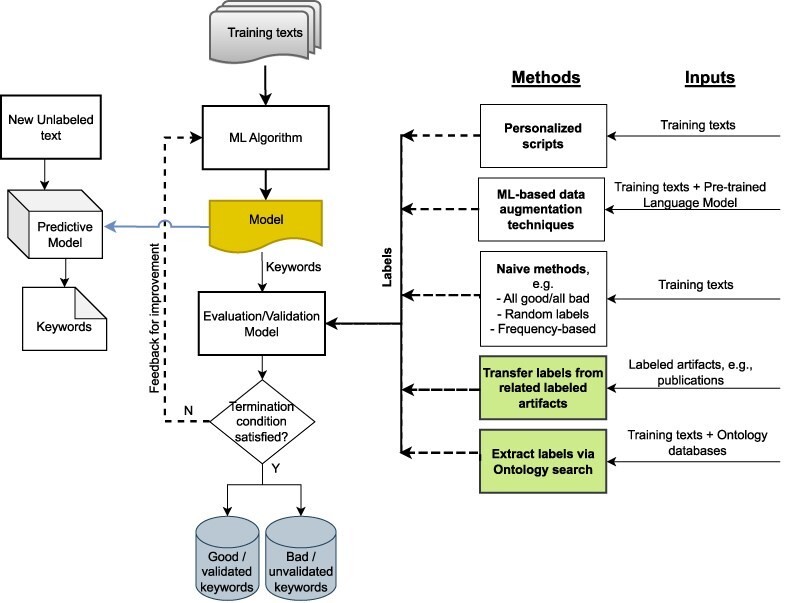
Potential approaches for validating ML-generated keywords for unlabeled texts when human labels are unavailable; our proposed approaches are shown in green.

High-throughput omics technologies such as genome sequencing produce a wealth of data in domains ranging from human health, biosurveillance, and environmental microbial ecology. However, these data frequently lack the metadata necessary for scientists to curate, search, integrate, and interpret these data appropriately. Manual labeling is time-consuming and error prone and is made more difficult by the paucity of gold-standard training sets, particularly in domains such as environmental science. Machine learning (ML)–based keyword extraction techniques have emerged as a potential solution to the challenge of automatically annotating scientific artifacts such as documents and images with the metadata necessary for enabling effective curation and search (1, 2). An example of ML-based keyword extraction is YAKE (3), which uses an unsupervised approach to generate keywords making use of surrounding document context. However, these ML-based approaches depend on the existence of text labels for either training the models (supervised methods) or validating the ML-generated metadata (unsupervised methods). Thus, applying these approaches to unlabeled scientific texts still requires the annotation of some of the documents before the quality of any ML-generated keywords can be determined (Figure 1). Given that manual annotation is tedious and not always feasible (4), the creation of annotated data sets and development of efficient automated annotation techniques for biomedical data sets are critical to ensuring effective data curation and accelerating scientific progress.

The biomedical community has produced a number of curated, high-quality annotation corpora such as CRAFT (5) that could potentially be leveraged to evaluate unsupervised keyword extraction methods. These corpora provide expert-curated linkages between spans of text and concepts and can be used to benchmark or train ML approaches to automating the linkages [a process called Concept Recognition (CR)]. However, these corpora focus on human health, covering human diseases, human and model organism anatomy, and drugs and do not cover many relevant concepts in environmental genomics. For example, consider a study of the response of microbiome communities in wetland ecosystems to rising sea levels and how they mediate methane emissions and carbon stocks (6). The study involves a number of concepts related to environmental genomics (e.g. rising sea levels and wetland ecosystems), which are not represented in biomedical corpora. Other examples include the study of root exudates in fabricated ecosystems under conditions of abiotic stress (7), which also requires concepts from corpora outside the biomedical domain (i.e. plant anatomy and genomics). An additional challenge arises from the disparate nature of text and metadata in modern team science and high-throughput multiomics studies. Text and metadata are often fragmented across the literature as well as different omics databases and sequencing facilities. In order to make maximal use of this information, it has to be intelligently curated and linked together such that information can be propagated.

The driving use case for our study is the vast amount of heterogeneous data generated by the Joint Genome Institute (JGI), a US Department of Energy (DOE) user facility with the mission to advance genomics in service of clean energy generation and environmental characterization and cleanup. The JGI has generated over 14 petabytes of data and is able to hone in on data sets via metadata search is crucial. Most JGI projects are initiated from a proposal, which includes rich textual information that can be indexed in search. These projects may then be linked to other information, such as publications, or paired omics data generated at other facilities. The metadata, while still complex, has more uniformity and structure than the diverse omics data that it describes and is thus more amenable to indexing. Still, curating all this information and indexing it with relevant concepts is challenging, especially as existing biomedical annotators are not trained with concepts directly relevant to the broad environmental problems being tackled by the JGI.

In this paper, we describe two automated text annotation techniques for the validation of ML keywords in order to enhance data curation and search. These approaches and their integration into a wide data science ecosystem is shown in Figure 1. The first approach, called “artifact linking”, exploits the relationships between different types of data artifacts related to the same research. Establishing direct relationships between artifacts is extremely powerful since it enables the transfer of text labels between the related labeled and unlabeled artifacts, making it a rich source of metadata. The second approach assigns labels to the unlabeled texts from controlled domain-specific ontologies. The technique exploits the fact that the most relevant text labels for scientific texts will contain domain-specific language that will be present in the ontologies relevant to that domain. The domain-specific relevance of the words and phrases in the unlabeled texts is determined via ontologies, with a frequency-based approach used to assign keyphrases to the texts.

Our work presents novel nonhuman, non-ML techniques for validating ML-generated metadata for unlabeled texts such as proposals (narrative descriptions of proposed hypothesis-driven research). Additionally, we compile parts of existing vocabularies and ontologies, primarily from the Open Bio Ontologies Library (8) into a novel application ontology called Biological and Environmental Resource Ontology (BERO), intended for applications such as environmental genomics.

Building on the foundations and infrastructure already in ScienceSearch (9), the approaches are applied to proposal texts from the JGI. Developing semiautomated text labeling techniques for JGI’s unlabeled data artifacts will enhance its search and indexing capabilities, thus potentially accelerating scientific discovery in genomics research. While the techniques presented are being demonstrated in the specific context of genomics, the techniques are general and can be applied to other scientific domains. Our semiautomated techniques for text labeling advance the state-of-the-art in keyphrase extraction in two ways.

1. We develop computationally inexpensive, nonhuman, non-learning-based text labeling approaches for the validation of ML-generated keywords for unlabeled texts. Once validated, the ML models can be applied to generate keywords for other documents in a given corpus for which the aforementioned annotations are not available or possible. Automated labeling removes the bottleneck of absent labels/keywords, one of the primary challenges associated with extracting relevant keywords with high accuracy (3).
2. Our approaches primarily exploit real available public (human) knowledge such as related scientific works and controlled vocabularies. This has several advantages, including being particularly suited to handling and exploiting the domain-specific nature of scientific texts.

The rest of the paper is organized as follows: the Background section provides important background information. The Methodology section presents our framework for automated label generation and details our two proposed approaches for automated label generation. It also presents the ML-based keyphrase evaluation approach employed, along with information about some of the other decisions made regarding label ranking and hyperparameter optimization. The results obtained for our proposed approaches are presented and discussed in the Results section, and some important observations about the results and methods proposed are discussed in the Discussion section. We conclude by presenting a review of related work in the Related Work section.

## Background

Our text labeling approaches have been developed and integrated into the ScienceSearch pipeline for automated metadata generation. In this section, we first present a summary of how the unlabeled JGI data artifacts that require labels are generated and stored. We then present a brief overview of the ScienceSearch infrastructure and how our work fits into the framework.

### JGI data generation and management

The sequencing and computational analysis capabilities offered by JGI lead to the generation of massive volumes of labeled and unlabeled data artifacts.

The use of JGI’s facilities begins with an application process, with submitted proposals evaluated by expert domain scientists as to their scientific significance and relevance to DOE science missions. As part of the JGI data management process, each approved proposal is assigned a unique integer Proposal ID that links projects and samples associated with the proposal. These archived proposals are the unlabeled scientific texts of interest in this work. For further context on the content and structure of the JGI proposals, we provide links to two examples of the proposals in our data set (https://jgi.doe.gov/wp-content/uploads/2022/09/example-JGI-Functional-Genomics-proposal.pdf, https://jgi.doe.gov/wp-content/uploads/2020/08/example-NI-Proposal-503942.pdf). The JGI proposals typically lack metadata related to their scientific contents, so developing techniques to automatically generate text labels is critical to facilitating search and indexing.

### ScienceSearch

ScienceSearch (9) is a generalized scientific search infrastructure that uses ML to capture metadata from data and surrounding artifacts. The ScienceSearch platform enhances search capabilities across several scientific domains including genomics, earth sciences, and microscopy. The search capabilities provided by the ScienceSearch infrastructure are critical to advancing scientific data exploration, allowing end users to search across different data artifact types (e.g. publications, proposals, file system paths, and images) and provide feedback on automatically generated tags. A key piece of the ScienceSearch pipeline is “SciKey,” the component responsible for automated metadata extraction.

### SciKey

**Figure 2.**
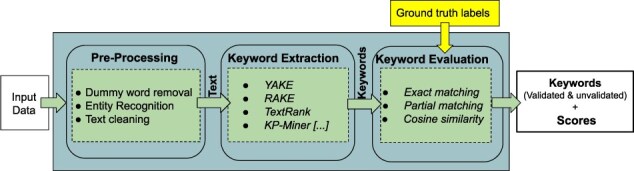
“SciKey’s” metadata generation pipeline and submodules.

“SciKey” (10) is a domain-specific, modular, customizable, keyword extraction pipeline that incorporates different natural language processing (NLP) extraction techniques for automatically generating keywords and keyphrases from scientific data sets. Figure 2 shows a simplified representation of the three submodules that make up “SciKey”: “preprocessing”, “keyword extraction” and “keyword evaluation.”

The “preprocessing” module prepares the raw input data for NLP ingestion. Scientific texts typically contain domain-specific, nonstandard text information such as abbreviations and acronyms, as well as nontext information such as numbers and punctuations. The preprocessing step contains submodules for (i) dummy word removal, (ii) named entity recognition, and (iii) text cleaning. The “keyword extraction” module generates keywords from the sanitized texts via NLP. “SciKey” offers a suite of unsupervised learning algorithms for NLP keyphrase extraction, including TextRank (11), RAKE (12), and YAKE (3). The “keyword evaluation” component of the “SciKey” pipeline computes quantitative metrics for the quality of the NLP keywords generated in the “keyword extraction” stage by comparing against a set of provided ground truths (i.e. labels) based on common information retrieval metrics such as cosine similarity and F-1 scores.

“SciKey” has been demonstrated to work well for labeled data sets (where ground truth labels are available). However, to take advantage of all the scientific information available, there is a need to extend these capabilities to unlabeled scientific data sets such as proposals and reports (where ground truth labels are not available). The techniques developed in this work are integrated into the “SciKey” pipeline (a key component of the keyphrase extraction step in this work) as a potential solution to this challenge.

## Methodology

**Figure 3.**
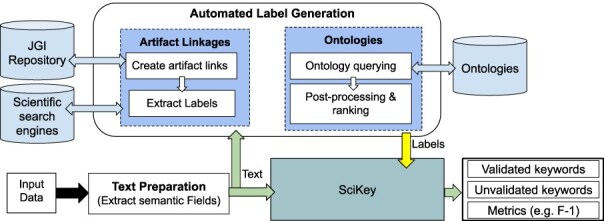
Overview of the automated labeling and metadata generation process. Built on top of the “SciKey” module, the automated label generation process (shown in the rounded rectangle) takes in a text blob containing semantic information and a set of labels that are used for ML-generated keyword validation. Based on these labels, “SciKey” outputs a set of validated ML keywords (i.e. “good” labels) and quantitative metrics reflecting keyword quality.

Figure 3 shows our automated label generation process and how it interacts with the “SciKey” pipeline.

The “text preparation” method extracts the semantic information from the raw input data and converts it into a format suitable for further NLP processing. The “automated label generation” component generates text labels from the processed outputs of the “text preparation” method. The method offers two techniques for generating text labels that exploit real, publicly available (human) knowledge such as related scientific works and controlled vocabularies. Finally, the generated text labels from the “automated label generation” step are passed to SciKey’s “keyword evaluation” submodule to assess the quality of the ML-generated keywords. The output from Scikey’s “keyword evaluation” is a set of quantitative metric scores that provide information about the quality of the ML-generated keywords when validated against the text labels. It should be noted that the automated label generation component and the first two SciKey modules (“pre-processing” and “keyword extraction”) can be run in parallel.

### Text preparation

The input data for the metadata extraction process are a raw, unlabeled data set of 2143 approved genomic research proposals available as comma-separated text (“.csv”). The “.csv” file contains a subset of all research proposed for investigation with the facilities available at the JGI over a period of 12 years (2009–20). The proposal file contains 35 metadata fields, as shown in Table 1, and can be classified as containing two types of information:

1. Fields containing document-related information about the proposal such as author institutions, proposal cycle, and completion date.
2. Fields containing semantic information related to the actual proposed research such as work description, justification, and community interest.

**Table 1.** List of metadata fields available

Semantic fields	Non-semantic fields	
	Proposal ID	Other Collaborators
	Type	All Collaborators
	Cycle	Survey Comment
Title	Focus Area	Contracts Office Contact for UA
Description	PI Name	Transfer Agreements Contact
Justification	PI Institution	Non-US Samples
Utilization	PI Email	Samples Regulatory Compliance
Community Interest	Co-PIs	Primary Funding Source
DOE Mission	Status	Funding Source Comment
Sample Preparation	Survey Choice	Completion Date
Summary of Work	All Institutions	Planned Publications
	Created At	Syn Bio Total KB
	Submitted At	Syn Bio Data Mining
	Date Approved	

The fields containing semantic information are shown in the left column.

While the document-related information is useful for provenance and data management purposes, they contain no information of semantic value and only introduce noise to the NLP process. Thus, for the label generation process, we only consider the fields containing semantic information; fields containing only document-related information (excluding the “proposal ID” field) were discarded. In the “text extraction” step, we identify and extract the columns containing relevant semantic information from each proposal. Of the 35 fields available in the raw proposal data set, eight fields were found to contain useful semantic information about the proposal: the “title, description, justification, community interest, summary of work, sample preparation, utilization,” and “DOE mission” fields.

The text strings contained in the eight fields are joined together to form a single text string for the next steps of the process.

### Automated label generation

We implemented two semiautomated techniques for generating quality text labels to be used in validating the ML-generated keywords: artifact linkages and ontology matching.

#### Artifact linkages

Linking unlabeled artifacts to directly related artifacts with known labels can provide a set of “derived labels” with which the unlabeled artifact can be associated and/or archived. In our case, we linked the unlabeled proposals to publication records; with each proposal inheriting the keywords from the publication(s), it could be associated with directly. An advantage of linking to publications is that since they contain human labels, the keywords transferred to the unlabeled texts as labels naturally incorporate the necessary semantic knowledge.

**Figure 4.**
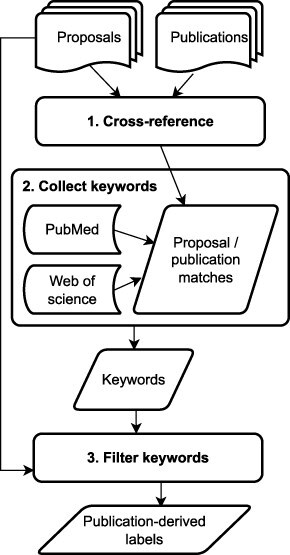
Flowchart of the artifact linkage process for label generation

Figure 4 presents a schematic representation of the artifact linkage process. For our use case, artifact linkage was achieved by cross-referencing the list of proposals against a curated list of publications. For the JGI problem, publications with JGI users and personnel as authors are linked to proposals that produced data or materials used in a given publication. These linkages are established by a combination of automatic assignments and manual curation by JGI staff. The proposal ID field, common to both proposals and publications, provided us with a way to link both types of artifacts.

We created direct links between 184 proposals and 337 publications by cross-referencing the full proposal set against a list of 488 JGI publications. The direct links were created by matching the unique “Proposal ID” field present in both types of data artifacts. These 184 proposals with associated publications were considered the training data/subset for the NLP keyphrase extraction model.

For each proposal, the keywords associated with the linked publications were then automatically curated from three online sources:

1. the “Author keywords” field from Web of Science (https://clarivate.com/webofsciencegroup/solutions/web-of-science/), a collection of keywords chosen by the author of the publication;
2. the “Keywords” field from PubMed (https://pubmed.ncbi.nlm.nih.gov/), also a collection of author-defined keywords for the publication; and
3. PubMed’s “MeSH terms” field, a collection of controlled vocabulary (medical subject headings) used to label articles topically by trained indexers.

Users will typically search for documents based on words expected to be present in its contents. Important keywords for a document will typically appear in the text body; words absent from a document’s content are less likely to be used to search for it. As such, we filtered out any keywords that did not appear in the proposal text. The remaining keywords obtained from these sources were assigned as training labels for the associated proposals (known as publication-derived labels henceforth).

The artifact linkage approach takes advantage of the relationships between artifacts; it is generally applicable and can be applied to any case where connections between labeled and unlabeled artifacts can be established in some way. This connection can be in the form of numeric tags (e.g. IDs, funding award numbers), strings (e.g. filenames), or even established manually (through interactions with the researchers). Additionally, in research environments, most unlabeled artifacts such as proposals and theses often lead to publications, which can serve as at least one recognized source of labels that is common to all scientific domains. Generally, different artifact types will be related to one another to varying degrees, and the level of relatedness of the artifacts being linked will have an impact on the strength and validity of the keyword association. In our case, there is a clear and direct link between the proposals for a work and the publications that arise out of it, and thus the derived labels are mostly expected to have a high degree of validity.

#### Ontology-based text annotation

We use expert-curated ontologies to annotate the proposals and identify words/phrases within each unlabeled proposal that are representative of the ideas and topics explored.

**Figure 5.**
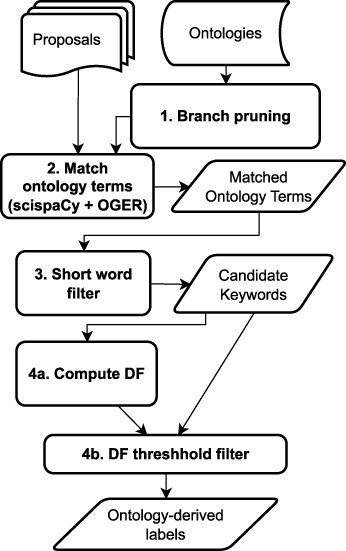
Flowchart of the ontology-based process for label generation

Figure 5 presents the key steps in the ontology-based labeling process. We identify potential metadata using ontologies via a two-stage process:

1. Identifying relevant phrases and keywords that are relevant to the domain (numbered 1–3 in Fig. 5).
2. Ranking the identified words and phrases in terms of importance to determine the proposal labels.

##### Identification of relevant phrases:

Written texts such as proposals are typically a mixture of both generic and domain-specific words and phrases. The goal of this step is to identify the list of all candidate labels for each proposal based on information curated by experts with domain knowledge. Ontologies predominantly contain domain-specific terms and phrases, and exploiting them allows us to identify which words present in the proposal domain experts believe are relevant in the context of the environmental genomics domain.

For our use case, to generate the candidate set of potential text labels for the 184 training proposals, we created an application ontology called BERO, consisting of the genomic, biological, and environmental subject areas and compiled a list of all the identified words and phrases ( “matched terms”). Table 2 provides the components ontologies used to create BERO. The ontologies are all open-source and publicly curated. The ontology is maintained on GitHub (https://github.com/berkeleybop/bero) and is available from BioPortal (https://bioportal.bioontology.org/ontologies/BERO). Identified in collaboration with domain experts, the ontologies cover the entire spectrum of focus areas and work investigated by the institute, including genomics, multiomics, bioinformatics, plants, organisms, and biological and environmental entities. We implement the ontology search and entity recognition step by embedding links to the ontologies into text processing and annotation tools specific to the biomedical domain. We use two tools:

1. OntoGene Entity Recognition (OGER) (13, 14), a biomedical named entity recognizer, and
2. scispaCy (15), a python package for biomedical text processing and Named Entity Recognition (NER).

**Table 2.** Components of BERO

Ontology	Focus/domain	References
Environment Ontology (EnvO)	Environmental features, habitats	(16, 17)
Gene Ontology (GO)	Biological functions and processes	(18, 19)
Chemical Entities of Biological Interest (ChEBI)	Molecular entities	(20)
National Centre for Biotechnology Information Taxonomy (NCBITaxon)	Organisms	(21)
Ontology of bioscientific data analysis and management (EDAM)	Bioscientific data and bioinformatics	(22)
Plant Ontology (PO)	Plant anatomy and genomics	(23, 24)
Molecular Process Ontology	Molecular processes	(25, 26)
Ontology for Biomedical Investigations (OBI)	Biomedical investigations	(27)
Phenotype And Trait Ontology (PATO)	Phynotype qualities	(28, 29)
Ontology of core ecological entities (ECOCORE)	Ecological entities	(31)

OGER and scispaCy parse the unlabeled text, query the various ontologies, and return an annotated list of matched terms. Part-of-speech tagging was done on the text with ScispaCy, allowing us to filter out parts of speech and matched terms that provided no information of semantic value (e.g. geographical locations).

An unguided ontology search would return every match found in the proposal texts without taking into account any sort of context, leading to some spurious word and phrase matches. At least two types of spurious matches were found to occur frequently:

1. cases where the ontologies matched words or phrases in the proposal exactly, but in the wrong context. This was found to be the case with words that have both domain-specific and general-purpose meaning (e.g. data, well, and sample);
2. cases in which words in the proposals were wrongly matched to acronyms for domain-specific phrases. This was found to occur with shorter words, especially when word stemming is applied. For example, an unguided search with the word “serv” (the stemmed version of the words serve and service) is a match (and acronym) for “simian endogeneous retrovirus type D, SERV” in NCBITaxon.

It is therefore important to implement search and downselection rules to minimize the likelihood of such spurious matches as candidate text labels. To achieve this and keep the size of the matched candidates manageable, two downselection rules were applied:

*Branch pruning*: Terms in the “branches” of the ontologies were selectively removed. Concepts in ontologies are typically categorized under a small number of subclasses called branches; in this case, we only considered a carefully curated selection of branches. This process, called “branch pruning,” was carried out before the ontology search (Step 1 in Fig. 5). The curation process was handled by a domain scientist familiar with the ontologies to ensure that only relevant ontology subclasses are retained. For example, within the Ontology of bioscientific data analysis and management (EDAM), of the branches “Topic,” “Operation,” “Data,” “Data Identifier,” and “Format,” only the “Topic” branch was retained because it includes broader interdisciplinary concepts from the biological domain. Similarly, for Phenotype And Trait Ontology, only the “physical quality” branch was retained.
*Exclude short words*: Words with less than three characters were dropped from the matched terms list. This rule was implemented such that acronyms are unaffected, so important keyphrases like DNA and Sulphur-Oxidizing Bacteria are retained. Short word removal is a postprocessing step after generating the ontology matches (Step 3 in Fig. 5).

The result of this step is the set of candidate labels present in the proposal that have been curated by experts with domain knowledge specific to the genomics field.

##### Ranking and filtering for ontology comparisons:

The output of the Ontology search process described earlier is a rich corpus of words per document. However, the returned words and phrases are unranked and have no associated scores to reflect the relative importance of the different words. Thus, we needed to devise an approach to rank the extracted labels. For this, we adopt the “document frequency” (DF), a measure of the rarity of a phrase in a given corpus. The DF for any phrase *w* is given by


$$ \textit{DF}(w)=\frac{\text{number of documents containing the term }w}{\text{total number of documents in corpus}}. $$

With this metric, the more unique a word is, the more important it is. The frequency of a word is inversely related to its value, with uniqueness treated as a proxy for importance.

The ontology-based annotation process typically results in a large number of word/phrase matches per document. However, documents are typically indexed by a limited set of keywords (typically under 20). Therefore, there needs to be a downselection of the number of labels based on their importance, as the number of labels can significantly impact the keyword evaluation process. To control the number of text labels to be considered per document, we tested different threshold limits for the DF across the corpus. For any specific threshold limit, only words with a DF score below that limit were considered as text labels for the documents. The threshold limit considered ranged from 1% (limiting to words occurring at most in two documents in the corpus) to 100% (no limit on the frequency of occurrence), with 1% arbitrarily selected as the baseline value. Setting threshold limits achieves two purposes:

1. it serves as a way to control the size/number of ontology-derived text labels used in the evaluation process and
2. it provides a way to independently assess the performance of the NLP algorithm on different types of ontology-derived text labels. The DF metric is a measure of how unique a keyphrase is, so low DF threshold limits allow us to investigate performance on document-specific labels, while high DF thresholds allow us to evaluate performance on both specific and generic labels. For the baseline case, we want the labels to be as document-specific as possible and hence the 1% threshold.

The matched terms which fall below the set threshold limit were treated as potential labels for the proposals (called ontology-derived labels henceforth). These labels are forwarded to “SciKey’s” keyword evaluation module.

The only requirement for the ontology-based approach is the availability of the domain-specific vocabularies. It is therefore generalizable to most ontology-aware domains (i.e. domains where collections of controlled vocabularies exist). We believe that there are a sufficiently similar usage and structure to most online ontologies that would allow our methods to apply to new domains. The applicability of the ontology-based approach is expected to cover a wide breadth of domains, from biology to environmental sciences to linguistics to computing.

The derived labels generated from the artifact linkage and ontology-based approaches proposed here are passed to “SciKey” as ground truths to validate the quality of the ML-generated keywords and tune the NLP models (Fig. 3).

### “SciKey” configuration for NLP keyphrase extraction and evaluation

As previously shown in Figure 3, the overall goal of the automated label generation process is to provide ground truth labels for the validation of ML-generated keywords from NLP algorithms. The ML keyword generation and validation process was done using the “SciKey” pipeline. Here, we summarize the ML keyword evaluation process with the SciKey pipeline (Figure 2) for our use case.

#### Preprocessing:

First, the text from the “text preparation” step was sanitized for NLP ingestion. Punctuations, URLs, numbers, and citations were removed using Regex-based approaches. We employed NER techniques and custom expert-curated lists to handle nonstandard scientific words and concepts. The sanitized text was then passed to the “keyphrase extraction” module.

#### Keyword extraction:

For our use case, we selected the YAKE NLP algorithm (3); an open-source Python implementation is available on GitHub (https://github.com/LIAAD/yake). While “SciKey” offers a variety of unsupervised learning algorithms for keyphrase extraction, we focus on YAKE because, for our use case, it produced the best results of the NLP algorithms we considered (YAKE, Rake, and KPMiner) in the keyword evaluation step based on exact matching metrics. However, any of the algorithms available in the pipeline could have been selected for the analysis. YAKE returns the extracted keywords for each proposal. These keywords (called machine-generated or YAKE-generated keywords henceforth) were forwarded to the keyword evaluation component of the ScienceSearch pipeline.

We used the training subset of 184 proposals to tune the YAKE parameters that control how the ML algorithm was applied or “hyperparameters:”

1. ***n*-gram size**. Longest contiguous sequence of *n*-words occurring in the text $\left({{\rm ngram}}\in[1, 2, 3]\right)$,
2. **Window size**. Sliding window size for YAKE $\left({\rm ws}\in[1,2,3]\right.)$
3. **Deduplication method**. Similarity metric for controlling deduplication (${\rm dedup}_{m} \in$ [Levenshtein distance, sequence matcher, Jaro-Winkler]).
4. **Deduplication threshold**. Allowable similarity between candidate ML keyphrases $({\rm dedup}_v\in[0.6, 0.7, 0.8, 0.9,$$0.95])$.

Hyperparameter tuning was done independently for the two sets of derived labels: for each top-*N* case, with *N* in $(5,10,20)$, we ran all 135 combinations of the four hyperparameters and chose the combination with the best F-1 scores for the training subset (Eq. 3), resulting in a different set hyperparameters in each case. These optimized models could then be used to generate keywords for the 1959 available proposals not in our training subset.

#### Keyword evaluation:

The quality of the ML-generated keywords generated in the previous stage were evaluated here using the “keyword evaluation” component of the SciKey pipeline. The keyword evaluation module takes two inputs (Fig. 2):

1. a list of machine-generated keywords (produced in the “keyword extraction” submodule of SciKey) and
2. a list of derived or ground truth labels for the ML labels to be compared against (produced by either the artifact linkage or ontology-based text annotation techniques described in the Automated label generation section).

We adopt the exact matching approach where the ML-generated keywords are compared against the derived labels for an exact string matching (2). For quantitative evaluation, we adopt the classical evaluation metrics used in information retrieval: precision, recall, and F-1 (2),


(1) $$ \text{precision} = \frac{\text{number of correctly matched keywords}}{\text{total number of extracted keywords}}, $$

(2) $$ \text{recall} = \frac{\text{number of correctly matched keywords}}{\text{total number of assigned/derived labels}}, $$

(3) $$ \text{F-1}=\frac{2}{\text{recall}^{-1}+\text{precision}^{-1}}. $$

Here, “correctly matched” means that the ML-generated keyword is also found in the list of derived labels. Stemming is applied to both the ML and derived labels using NLTK’s PorterStemmer (https://www.nltk.org/_modules/nltk/stem/porter.html) to eliminate spurious mismatches. We generate the metrics for top-*N* ranked (by YAKE) keywords, with *N* being 5, 10, or 20.

### Summary

We have presented two techniques by which labels may be automatically generated for unlabeled scientific texts. Once the semantically important section of the unlabeled text is identified and extracted, text labels can be (i) transferred over from generated from directly related research or (ii) generated using expert-curated ontologies. The derived labels generated by these techniques can be treated as ground truth labels for evaluating the quality of the ML-generated keywords (e.g. from the “SciKey” pipeline). The “automated label generation” pipeline is unique, providing us with alternative ways to evaluate the performance of an ML model and validate the quality of the ML-generated keywords without the dependence on direct manual human labeling. The validated model can, in turn, be used to generate keywords for the rest of the corpus with a good degree of confidence. For example, while we are only able to create artifact linkages for a subset of our proposal corpus (184 out of 2143), the validated ML model we train can be used to generate keywords for the rest of the proposals. Thus, for scientific texts, exploiting the domain knowledge already available via ontologies and artifact linkages for the labeling are a first step toward the automation of keyword extraction.

## Results

This work proposes two techniques—artifact linkages and ontologies—for validating ML-generated keywords for unlabeled scientific texts. In this section, we assess the characteristics and quality of both techniques. First, we present an analysis of the text labels generated by both approaches. We follow this with an analysis of the YAKE ML algorithm with respect to both sets of derived labels (NLP performance section).

These results were generated using Python 3.8 on a Thinkpad X1 Extreme running Windows 10 Pro version 21H2 with 32GB RAM and an Intel i7 processor.

### Analysis of derived labels

Both approaches derived labels for 184 scientific proposals.

#### Publication-derived labels

**Figure 6.**
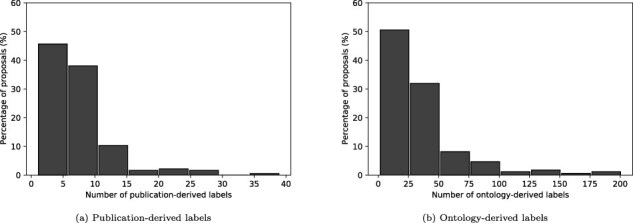
Distribution of derived labels.

A total of 1294 labels were obtained from the keywords of publications associated with the proposals, as described in Artifact linkages. Figure 6a shows the distribution of the number of labels obtained per proposal. Most of the proposals (83%) have 10 or fewer labels, while roughly 4% of the proposals have over 20.

An assessment of the lengths of the derived labels (Table 3) shows that all the labels had between one word (unigrams) and three words (trigrams). About 85% of the labels are unigrams (one word), and less than 2% are trigrams (three words). The result is weighted more toward unigrams which is in line with other works such as Campos et. al. (30), who report averages of 47%, 34%, and 13% for unigrams, bigrams, and trigrams, respectively.

**Table 3.** “*n*-gram” summary for derived labels

	Publication-derived		Ontology-derived	
	No. of labels	Percent	No. of labels	Percent
1-gram	1094	84.5	3360	62.7
2-gram	183	14.1	1810	33.7
3-gram	17	1.3	171	3.2
4-gram			22	0.4
Total	1294		5363	

The 1294 labels contain 520 distinct words or phrases (Table 4). Frequency analysis shows that $\approx 70\%$ of the labels are associated with one specific proposal, indicating that the labels generated for each proposal tend to be fairly unique. Over 80% of the labels are associated with two proposals at most (i.e. 1% of our training corpus). Of the 520 unique labels, nine (1.7%) occur in over 10% of the corpus. Table 5 shows examples of some of the labels found. The most frequent labels are typical and representative of the subject area, with the most common keyword, “genom” (stemmed version of genome and genomic), occurring in over 40% of the proposals. The labels for each document are also semantically different in meaning and context; an evaluation of the pair-wise cosine similarity scores of the labels of each proposal (Table 6) shows that most of the label pairs are semantically unrelated (both the average and median scores are close to zero), with over 90% of the label pairs falling between -0.15 and 0.59.

**Table 4.** Summary of frequency of occurrence of all derived labels (after stemming) in 184 proposals.

Frequency	Publication-derived		Ontology derived	
	No. of labels	Percent	No. of labels	Percent
1	360	69.2	3153	80.2
2	66	12.7	544	13.8
3	29	5.6	151	3.8
4	15	2.9	56	1.4
5	13	2.5	12	0.3
6–10	17	3.3	17	0.4
11–20	15	2.9	0	0
> 20	5	1.0	0	0
Total	520		3933	

**Table 5.** Ten most and least common publication-derived labels (after stemming) by frequency

Most common	“genom”: 76, “bacteria”: 38, “divers”: 36, “metagenom”: 33, “sequenc”: 27, “gene”: 20, “carbon”: 20, “dna”: 19, “rna”: 19, “soil”: 16
Least common	“aromat compound”: 1, “valor”: 1, “saccharum”: 1, “hybrid”: 1, “haplotyp”: 1, “phylogenet analysi”: 1, “polyploidi”: 1, “spontaneum”: 1, “sugarcan”: 1, “glycin betain”: 1

**Table 6.** Pair-wise cosine similarity statistics and examples.

	Publication-derived	Ontology derived
Mean	0.0645	0.0229
Standard deviation	0.02079	0.1430
min.	-0.3461	-0.4306
5%	-0.1575	-0.1626
50%	0.0143	0.0061
95%	0.5910	0.2742
max.	0.9903	0.9930
Top-3 most similar	“cassava-manihot,” “archea-bacteria,” “soil-peatland”	“willow-salix,” “cycad-macrozamia,” “sand-quartz”

**Figure 7.**
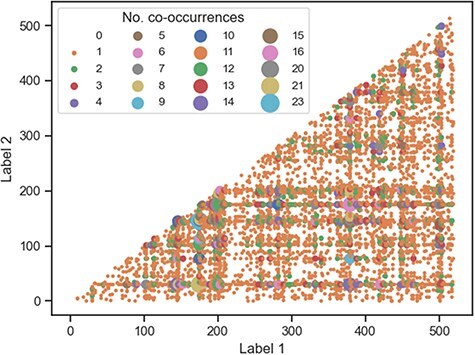
Co-occurrence plot for all publication-derived labels. Most of the labels either do not co-occur at all (white space) or co-occur once (small orange circles).

To gain a better understanding of how the labels are linked to each other across documents, we investigated the frequency of co-occurrence of all 520 unique publication-derived labels (Figure 7). Most label pairs either never co-occur or only co-occur in the corpus once—of the 1 34 940 potential co-occurrence pairs, only 681 pairs ($\lt1\%$) occur more than once. This suggests that the combination of labels for any specific document is unique and not redundant. As expected, the most frequent co-occurring label pairs are those made up of the most common labels (Table 5), with the top five pairs being “genom-metagenom” (23), “genom-divers” (21), “genom-sequenc” (20), “genom-bacteria” (16), and “bacteria-metagenom” (15).

#### Ontology-derived labels

A total of 5363 ontology matches were found for the 184 proposals, using the methodology described in the Ontology-based text annotation section. Figure 6b shows the distribution of the number of labels obtained per proposal. Most of the documents ($\approx76\%$) have 50 or fewer labels, while roughly 4% of the proposals have over 100. An analysis of the ontology terms (Table 3) reveals that the labels are more evenly distributed than in the publication-derived label case, with bigrams making up just over a third of the labels. Again, very few labels have more than two words. We find that a large proportion of the bigrams and trigram labels are simple combinations of the unigram labels identified (e.g. whole-genome sequencing and soil metagenome).

The predominance of unigrams and bigrams in both sets of derived labels is in agreement with the literature, which suggests that people rarely use more than three terms to describe a given subject (33).

The 5363 ontology matches obtained contain 3933 distinct words or phrases (Table 4). Most of the matches are unique, with over 80% associated with one specific proposal. As with the publication-derived case, the pair-wise cosine similarity scores show that the labels for each document are also semantically distinct in meaning, with over 90% of the label pairs falling between -0.17 and 0.28 (Table 6). Where synonyms of species or important concepts are present in the document (e.g. willow/salix, cycad/macrozamia), both terms are often identified as document labels.

### NLP performance

We ran the YAKE NLP algorithm and evaluated the generated keywords as described in “SciKey” configuration for NLP keyphrase extraction and evaluation section. This section presents the performance against the publication and ontology-derived labels.

#### Publication-derived labels

Table 7a shows the results of evaluating YAKE against the publication-derived labels. The F-1 scores are similar to recently published results on keyphrase extraction for popular author-labeled scientific data sets such as Krapavin, Semieval2010, NUS, and Inspec (Table 8). Thus, we are able to obtain good metadata for the proposals (compared to the state-of-the-art). The best results were obtained at F-1$@ 10$, which indicates that $@10$ provides the best balance between increasing the number of correct matches overall (recall) and keeping the number of false positives low (precision).

**Table 7.** YAKE precision (P), recall (R), and F-1 scores on derived labels.

	P	R	F-1
**(a) Publication-derived**			
@5	0.255	0.246	0.250
@10	0.200	0.340	0.252
@20	0.149	0.438	0.222
**(b) Ontology-derived for a DF threshold of 1%**			
@5	0.094	0.105	0.099
@10	0.079	0.138	0.100
@20	0.068	0.163	0.096

**Table 8.** Best F-1 scores reported in (2) for some classical scientific collections using the exact matching approach.

Data set	Type	F-1 @ 10	F-1 @ 20
NUS	Full-text papers	0.259	0.243
Krapivin	Full-text papers	0.190	0.161
Semeval2010	Full-text papers	0.208	0.219
Inspec	Paper abstracts	0.278	0.295
**Our work (w/YAKE)**	**Proposals**	**0.252**	**0.222**

For comparison, our results are shown in bold on the last row.

Table 9 summarizes the optimal YAKE hyperparameter values for the three F-1 cases. The optimal YAKE settings are similar in all cases; the only difference occurs in the choice of window size for the F-1$@5$ case. The optimal “ngram” size of one is not surprising given the heavy bias of the derived labels toward unigrams, as highlighted in the Publication-derived labels section.

**Table 9.** Optimal YAKE hyperparameters with publication-derived labels

	${\rm ws}$	${\rm ngram}$	dedup_*m*_	dedup_*v*_
@5	3	1	Levenshtein distance, sequence matcher	0.9
@10	2	1	Sequence matcher	0.9
@20	2	1	Sequence matcher	0.9

Sample results from the first three proposal documents (Table 10) illustrate some aspects of the matches that are not obvious from the quantitative metrics. All three of the examples show that we can match both general (e.g. “genom”) and very specific keyphrases (“amanita”, “desulfitobacterium”). The third document shows that while the ML algorithm is unable to match the trigram keyphrase “evolut of symbiosi” since “of” is a stopword, it does match the component words “evolut” and “symbiosi”. Thus, while some of the keyphrases suggested by the ML algorithm are not present in the exact form listed in the derived labels and are penalized by the exact matching metric, they still represent valid keywords for the documents and should not be discarded. Furthermore, a cursory comparison of the keywords to the proposal titles reveals that some potential representative keywords present in the ML-generated keyphrase list are absent from the derived label list. For example, the keyword “thersii” refers to the specific strain of Amanita under investigation and is thus an important keyword; however, the quantitative results do not capture this since it is not present in the list of labels. This suggests that the quality of the metadata obtained for the proposals extends beyond just the keywords matched and quantitative metrics and reflects one of the well-known challenges of extracting relevant keywords: the high number of candidate keywords that can be generated from any single text makes it difficult to position the most important ones at the top (3).

**Table 10.** Examples of derived and ML-generated keyphrases obtained with YAKE for F-1@10.

Title	Publication-derived labels	ML-generated keyphrases
1 – *Halorespiring Firmicutes: Exploring genomic plasticity of closely related dedicated degraders with diverging ecophysiological features and bioremediating capacity*	**“desulfitobacterium,” “genom”**	**“desulfitobacterium,”** strain, isol, environment, sequenc, dehalobact, **“genom,”** halorespir, bacteria, degrad
2 – *Resources for study of diversity and divergence in Sorghum, a C4 cereal model*	polymorph, rice, evolut, diverg, trait, gene, **“genom,” “sorghum”**	function, saccharum, crop, saccharina, sequenc, grass, variat, **“genom,” “sorghum,”** divers
3 – *Sequencing the genome of the basidiomycete fungus Amanita thiersii, a cellulose degrading fungus in an ectomycorrhizal genus*	compar genom, evolut of symbiosi, **“sequenc,” “genom,”** evolutionari, **“amanita”**	symbiosi, ectomycorrhiz, evolut, thiersii, saprotroph, speci, **“sequenc,” “genom,”** genu, **“amanita”**

While the F-1 score is highest for the top-10 keywords, the choice of metrics for a search or indexing task may depend on the specific use case and priorities. In some situations, prioritizing precision may be more appropriate, while in others, maximizing recall is more important. For instance, during research conceptualization, finding all relevant literature on a given subject is crucial, and in such cases, a higher recall may be more desirable. Our results show that predicting the top-20 keywords provides the highest recall, retrieving approximately 44% of the derived keyphrases (Table 7a). However, if noise in the ML results is a concern, precision may be a more appropriate metric to focus on, and F-1@5 represents the best option, ensuring that 26% of the generated ML keywords are relevant.

#### Ontology-derived labels

Table 7b presents the results obtained for the JGI proposals with YAKE for the baseline DF threshold of 1%. The F-1 scores obtained are slightly worse than those obtained with the publication-derived labels (Table 7a) as well as scientific data sets in the literature. This may be attributed to two reasons. First, ontology searches do not account for semantic and contextual information, so while we find many ontology matches, the matches also contain a lot of noise: matches that have domain-specific meaning but are not contextually important. For example, words like “co-culture,” “food,” “annotation,” “assay,” and “human” are present as derived labels because they have biological relevance and are thus present in the ontologies, but they have little value as keywords for the proposals. The absence of ranking for the ontology labels means that these contextually irrelevant matches are difficult to separate out automatically. The NLP algorithm is thus unfairly evaluated because the performance metrics (recall and F-1) are computed based on an inflated number of false negatives. Second, ontologies often have additional synonyms for a concept, in addition to the primary label, and can represent the same concept at different levels of abstraction. For example, “dehalobacterium” and “dehalobacterium sp.” exist as different entries in one ontology database and are thus treated as separate labels despite referring to the same bacteria genus. While a match to either of these terms is sufficient in reality, the evaluation technique penalizes labels not matched exactly (i.e. as false negative), leading to lower F-1 scores.

To understand the sensitivity of the results to the DF threshold value, we retrained the ML model with different threshold values from 1 to 100% and computed the F-1 scores (Table 11). The best F-1 scores are obtained when the NLP algorithm generates only 10 keywords; further increases in the number of keywords only worsen the NLP algorithm performance. This indicates that keywords that are most unique to each of the proposal texts are ranked high and returned early by the NLP algorithm. The analysis also revealed that precision and recall are influenced differently by the selected threshold and number of keywords. Precision increases proportionally with the threshold but decreases with the higher number of keywords. On the other hand, while the recall increases with the number of keywords, it is relatively unaffected by the selected threshold.

**Table 11.** YAKE performance on ontology-derived keywords for DF different thresholds.

		F-1		
Threshold (%)	Average number ontology keywords (prestemming)	@5	@10	@20
1.0	21.64	0.099	**0.100**	0.096
2.0	23.50	0.102	**0.105**	0.101
5.0	27.20	0.107	**0.112**	0.108
10.0	28.48	0.108	**0.113**	0.110
20.0	29.39	0.108	**0.113**	0.110
25.0	29.55	0.108	**0.113**	0.111
50.0	29.88	0.109	**0.115**	0.112
100	29.93	0.109	**0.115**	0.112

The highest F-1 score for each threshold is bolded.

In all the F-1@10 cases, the best results were obtained in YAKE with setting ngram = 2. This marks a change from the optimal setting of ngram = 1 obtained with the publication-derived labels (NLP performance section) and reflects the higher fraction of bigrams in the ontology-derived labels set (Table 3).

Table 12 presents the keywords obtained for the first three proposal documents for F-1@10 and threshold = 1%. A qualitative comparison of the generated keywords shows that we are able to find unigram and bigram keyphrases specific to each document (e.g. “johnson grass, saccharina”). However, keyphrases that are likely to be more generic (e.g. “genome”) are not validated because they are absent from the ontology-derived label set (eliminated by the frequency filter). For example, the ML-generated keyword “cell wall” is present in the full list of labels for the second proposal; however it occurs in five documents (2.7%) and is therefore not considered in this case. It is worthwhile to note that some of the validated keywords match those found in the publication-derived labels case.

**Table 12.** Examples of ML-generated keyphrases obtained with YAKE @ 10; threshold=1%.

Title	Derived labels (from ontologies)	ML-generated keyphrases
1 – *Halorespiring Firmicutes: Exploring genomic plasticity of closely related dedicated degraders with diverging ecophysiological features and bioremediating capacity*	(36^a^) - halogen compound, dehalogen, clostridium, dehalobact restrictu, **dehalobact**, dehalobact sp., verrucomicrobium, gram-posit bacteria, sedimentibact, sedimentibact sp., **desulfitobacterium**, desulfitobacterium hafniens tcp-a, desulfitobacterium hafniens dp7, desulfitobacterium hafniens, desulfitobacterium metallireducen, threat, reduct, adapt	plastic, elucid genom, halorespir, strain desulfitobacterium, strain, **dehalobact**, genom sequenc, genom, **desulfitobacterium**, sequenc
2 – *Resources for study of diversity and divergence in Sorghum, a C4 cereal model*	(16) - high temperatur, fossil-fuel, population-genet, demograph, strength, mutat rate, bac, gene order, saccharum, water suppli, **johnson grass**, motiv, **sorghum**, **saccharina**, bank, attract	saccharina function, sorghum genom, sorghum genu, **johnson grass**, **sorghum**, **saccharina**, cell wall, genom, function genom, sorghum sorghum
3 – *Sequencing the genome of the basidiomycete fungus Amanita thiersii, a cellulose degrading fungus in an ectomycorrhizal genus*	(7) - decompos, cellulos degrad, amanita: thiersii, laccaria, **amanita thiersii**, laccaria bicolor, isotop	thiersii genom, genu amanita, compar genom, **amanita thiersii**, ectomycorrhiz genu, heather hallen, genom sequenc, amanita speci, genom, ectomycorrhiz symbiosi

The total number of ontology-derived labels below the threshold is shown in brackets, while the matched keywords are in bold. aOnly half of the 36 ontology-derived labels are shown here.

The labels for the first document highlight the key challenges with the ontology-based approach: we have at least three variants of *Desulfitobacterium hafniense*, while some of the labels would be poor representatives for the proposals (e.g. threat and adapt). Further postprocessing of the labels before ML would therefore be beneficial in improving the quality of the results.

## Discussion

### Strength of association and validity of derived labels.

The ML-generated keywords have been evaluated using keyphrases not directly associated with the texts, thus requiring an assessment of the validity of the derived labels. The publication-derived labels are from published works that directly leverage data or materials produced through the linked proposals. Thus, these labels are expected to be strongly representative of the proposals, but this may not always be the case. There are two specific conditions where the association may be weak: when the products of the proposal are used but do not play a significant role in the publication or when the publication covers topics or concepts that differ from those originally stated in the proposal. In such cases, the publication-derived keywords may not accurately reflect the contents of a given proposal. Generally, however, we expect the publication-derived keywords to be representative of the proposals. The ontology-derived keyphrases are extracted directly from the proposal texts, making them strongly associated. However, with a frequency-based ranking approach for the ontology-derived labels, questions remain over whether the frequency-based ranks assigned to the individual keyphrases accurately reflect their actual contextual and semantic importance as document labels. The publication-derived keyphrases are considered the more reliable of the two sources due to the semantic and contextual information incorporated by humans into the keyphrase selection process (expert annotators in the case of MESH terms assigned by PubMed). Developing a ranking approach that takes into account the contexts of the extracted keyphrases would help increase confidence in the ontology-derived labels.

### Quality and human-in-the-loop for derived labels.

Assigning labels and/or keywords to any document is inherently subjective. User-specified labels are considered the gold standard for text summarization (32); however, even with that approach, not all the potentially correct keywords are assigned by users. Researchers typically pick keywords in *ad hoc* ways that are far from optimal and usually biased (33), and some phrases that are unsuitable as keywords are often included. We encountered the same challenge with the labels generated via artifact linkages (i.e. the publication-derived labels): the ML algorithm found several good candidate keywords that were absent from the derived labels list (e.g. “thiersii,” “halorespiring”). Thus, a “post-ML” step of human-in-the-loop keyphrase validation of the ML-generated keywords will be beneficial for improving the quality of the publication-derived labeling approach and ensuring that good keywords are not lost.

As expected, the ontology search returns significantly more derived keywords per document than the artifact linkage approach. However, it also returns a few generic, low-quality, nondomain-specific terms (e.g. “threat,” “strength,” and “attract”). With the ontology-derived labels, the challenge is the opposite of that described earlier with artifact linkage, with potential candidate keywords possibly being lost at the filtering stage. Thus, with the ontology-CR based approach, human-in-the-loop intervention to improve keyword quality will be most beneficial as a “pre-ML” step.

### Generality of ML-generated keywords.

Our results show that the ML-generated keywords for both cases contain some keywords too generic to be semantically or contextually useful (e.g. “grass,” “diversity,” and “divergence”). These could be eliminated by improving the stopword list.

Regarding the validated (i.e matched) keywords, the results show that we can match both generic and document-specific keywords irrespective of the derived labels source. However, the ontology-based approach has the advantage of having a hyperparameter (i.e. the threshold limit) that controls the uniqueness of the validated keywords. This is very useful for eliminating words like “genome” and “sequencing” that, while domain-specific, will occur frequently in a genomic corpus.

### Sensitivity to stopwords.

The results observed with the ontology-derived labels were found to be very sensitive to the list of stopwords. Some of the words generally found in scientific texts such as “observations,”, “findings,” and “field” have domain-specific connotations in the field of genomics and therefore have entries in biological and biomedical ontologies. Such words need to be handled explicitly to avoid their inclusion as potential ontology-derived labels, and the most logical approach is to include them in the list of stopwords. Thus, careful curation of the stopwords list is crucial if the ontology-based labeling process is to be adopted. Since the publication-derived labels are human-curated, such words are less likely to exist in the keyword list.

### Performance.

The results show that we perform better in validating the labels generated via artifact linkages (i.e. the publication-derived labels generated as described in the Artifact linkages section). This occurs because the publication-derived labels are fewer but of higher quality. A total of 44% of the ML-generated keywords were validated via the artifact-linkage approach, compared to the 23% validated with the ontology-derived labels. The two cases require different *n*-gram settings, each reflecting the “ngram” distribution of their derived labels. Both approaches have some validated keywords in common; such keywords are expected to be good representative summarizations of the proposal texts.

### Comparison against current state-of-the-art.

To demonstrate the benefits of our annotation methods, we compare our results against a state-of-the-art technique for text annotation, which involves the utilization of pretrained language models.

Table 13 shows the labels generated for three documents (same proposals shown in Tables 10 and 12) by two state-of-the-art language models: Vicuna-7B (34) and LLAMA2-13B (35). The language models were run on graphics processing unit (GPU) nodes on the National Energy Research Scientific Computing Center (NERSC) Perlmutter supercomputer; details about the exact prompt may be found in the Appendix A: Generative model prompt for label generation section. First, we observe that Vicuna-7B fails to generate labels for the first two documents because the proposal lengths exceed the maximum context window size (2048 tokens). This highlights one of the challenges with using language models for annotation: while they work well for short documents such as abstracts, they can often fail for longer documents (although techniques such as chunking can be used to get around this). The approaches we present in this work, which are unaffected by the length of the unlabeled texts, provide an alternative in such cases.

**Table 13.** Comparison against two state-of-the-art language models.

	Vicuna-7B		LLAMA2-13B	
Doc No.	Labels	% match	Labels	% match
1	-	-	**dehalogen, genom, adapt, pollut, *bacteria, sequenc***, halorespir, microdivers, environment, biotechnolog	60%
2	-	-	**diverg, saccharina, sorghum, evolut, genom,** divers, C4 photosynthesi, biofeedstock, variat, select	50%
3	**genom, compar genom, laccaria bicolor**, ***cellulos, symbiosi,*** fungu, ectomycorrhiz, saprotroph, phylogenet, carbon metabol	50%	**genom, amanita, thiersii**, ***symbiosi, cellulos,*** saprotroph, ectomycorrhiz, carbon, metabol, biofuel	50%
Inference time (s)	17.3		26.4	

The bold words are exact matches to either the publication- or ontology-derived labels, while the words in italics are partial matches. Vicuna-7B fails for the first two documents due to context window restrictions.

A comparison of the labels produced by the language models against our methods shows that our non-ML-based approaches find over 50% of the labels suggested by the language models. This indicates that there is fairly good agreement between our approaches and the state-of-the-art; the remaining portion of unmatched terms generated by the language model would require validation themselves. While Vicuna-7B and LLAMA2-13B find some good labels not suggested by our approaches (e.g. “halorespiration, biofeedstock”), they also miss other quality labels found by our methods (e.g. “desulfitobacterium, Johnson grass”). In cases where the matches are partial, we find that this is because our proposed approaches tend to find more specific terms than the language models (e.g. “gram-positive bacteria” and “cellulose degradation” from our methods vs. “bacteria” and “cellulose” from the language models). In such cases, we believe that our more specific labels are more useful. Overall, we find that the quality of the labels generated by our approaches are at least as good as those obtained with the pretrained language models. Our methods achieve this at significantly lower computational and financial costs. Language models have large memory (i.e. RAM) requirements, often necessitate the need for special hardware to run efficiently locally (e.g. GPUs), and may incur financial costs at inference time (e.g. GPT models); our proposed approaches do not require anything more than a desktop machine (no GPUs required).

An additional consideration here is that the use of ML techniques to generate the labels for evaluating the performance of another ML algorithm is conceptually circular; validating one ML algorithm with another lacks independent verification, raising questions around the credibility of the outputs. A key strength of our labeling approaches, which do not rely on machine learning, is that rather than depending on ML models to validate themselves or one another, we identify human-generated labels for validating the output of ML models. This is essential for transparency and to eliminate bias, given the black-box nature of current language models. Thus, our methods achieve comparable performance to state-of-the-art approaches while significantly requiring significantly lower computational costs. Notably, our approaches retain the crucial element of human-like validation, support transparency, and demonstrate enhanced suitability for longer documents and privacy-sensitive use cases (e.g. with JGI proposals which are not public).

We acknowledge that the language models can be used to generate keywords for the documents directly, rather than to generate labels for training an NLP model like YAKE. The keywords we used to validate our YAKE model also could arguably be used to train or provide context to more modern models instead. However, the inference process with this approach would be both computationally expensive and time consuming and thus would not scale well to a large corpus. For example, running YAKE for all 184 documents on a desktop takes 11.2 s (i.e. 0.06 s per document); in comparison, generating keywords from Vicuna-7B and LLAMA-13B requires 5–9 s per document on a 40GB RAM GPU supercomputer node.

### Generality of proposed text labeling approaches.

While this work demonstrates the applicability of the text labeling approaches proposed in the genomics domain, we believe that the techniques are generalizable to other scientific domains.

The artifact linkage approach comprises three main steps (Figure 4): (i) document cross-referencing, (ii) keyword collection for linked artifacts from online scientific databases, and (iii) keyword filtering. The cross-referencing step involves the use of additional input in the form of labeled artifacts that can be linked to the unlabeled input texts. Most unlabeled scientific texts can be directly linked to specific scientific research projects that produce other labeled artifacts, such as publications or DOE technical reports. However, while JGI has implemented workflows and practices for associating publications with user proposals, doing so may be a nontrivial and expensive process, and the information required to create these links reliably may not always be available. Furthermore, the criteria for associating research artifacts like publications and proposals could be different at other organizations than those used by JGI. Having access to an existing corpus or a means for producing similar artifact linkages is thus a precondition for this approach. Provided that an organization can satisfy this precondition, a new application will simply require custom scripts based on how the artifact links are created and the systems involved. The resulting information remains the same as that leveraged in the present study, making the cross-referencing step feasibly generalizable to other domains. Additionally, the cross-referencing step only requires enough information that allows the labeled text to be found (e.g. title, DOI, or PMID), not the full document itself. The second stage of the artifact linkage process is generic and can be applied out of the box if the labeled documents are on PubMed or Web of Science. For other online databases (e.g. Scopus), custom methods specific to parsing information for those databases will be required, but the general concepts will remain the same. The keywords are extracted from the metadata that third-party databases have indexed about the articles, rather than from the articles themselves. The approach, therefore, avoids paywall bottlenecks. The final keyword filtering stage utilizes Regex string matching—it is generic and requires no additional customization to use. Thus, while the process of identifying the link between the labeled and unlabeled artifact may be bespoke based on how connections between the artifacts are established, the other steps are generic.

The ontology-based approach for the text label generation consists of four main steps, as shown in Figure 5: branch pruning, ontology term matching, short word filter, and threshold filtering. To use the ontology-based approach in a different application area, the first stage of the pipeline needs to be modified to fit the specific domain of interest by incorporating and integrating the links to the appropriate domain-specific ontologies into an NLP tool. Most scientific application areas are ontology-aware (36), and there is a reasonable similarity in usage and structure to most online ontologies that would allow the proposed methods to be applied directly to those domains. Thus, while the ontology embedding stage requires some domain-specific customization, the requirements, information, and tools required are the same irrespective of the domain, making the approach generalizable. The remaining stages of the pipeline require no modifications or domain-specific customizations and can be implemented essentially as described in this work.

An example use case where these methods could be applied beyond our JGI use case is in enhancing the accessibility of the wide variety of social science research data sets available through the Inter-University Consortium for Political and Social Research (ICPSR (https://www.icpsr.umich.edu/web/pages/)). ICPSR curates and maintains linkages between its hosted data sets and scientific literature and provides public unlabeled, unstructured text information describing the hosted data sets (https://www.icpsr.umich.edu/web/pages/ICPSR/citations/). Exploiting the publication-data set linkages already available would provide a way to generate labels for the unstructured text contents of the data set descriptions. Similarly, our ontology-based approach could be applied to generate labels for the data set descriptions. The social science domain is ontology-aware; for example, the report by National Academy of Sciences, Engineering and Medicine (37) provides a list of some of the existing ontologies in the behavioral sciences. Ontologies representing different social science subfields of interest (e.g. behavioral science, education, aging, and criminal justice) would need to be identified and curated into a single ontology. In the absence of domain-specific NER tools, the ontologies can be embedded directly into general-purpose NER tools such as SpaCy(38). Once this is done, our technique could be applied directly to generate labels for the data set descriptions.

## Related Work

In this section, we present a brief review of works related to keyword extraction and initiatives to automatically label or augment data for NLP.

### Keyword extraction:

There is a large body of work focused on addressing the longstanding problem of extracting relevant keywords from scientific data. NLP provides the capacity to understand (39,40), summarize (41), paraphrase (42), categorize (43), and extract key terms and phrases (44) from scientific texts. Supervised, semisupervised, and unsupervised ML methods have all been applied to keyphrase extraction problems to varying degrees of success (45) and (46) provide an extensive review of the current state-of-the-art. Within the context of keyphrase extraction, the approach we adopt in this work (via SciKey) is unsupervised, with additional capabilities for incorporating domain-specific text processing, named entity recognition, and frequency analysis.

### Automated text labeling and data augmentation:

There have been numerous efforts to automatically label and/or improve the training data for these NLP problems. The traditional approach to addressing the lack of labels has been to focus on generative data augmentation using pretrained or large language models (47–50). With this approach, a small amount of labeled data is used to train a language model that produces labeled synthetic data for supervised NLP tasks, with the synthetic data used to train the final NLP model. For example, several researchers have adopted this approach to perform data augmentation for text classification by fine-tuning a language model to synthesize new inputs *x* for a given label *y* (47, 48). Similarly, data augmentation techniques such as “AugGPT” (51), “GPT3Mix” (52), “LAMBADA” (53), and “DARE” (50) generate synthetic training data for supervised learning and text classification by fine-tuning neural network models such as GPT-2 (54) and GPT-3 (55). More recently, the concept of “zero-label language learning” for NLPs has been explored to eliminate the need for fine-tuning the pretrained language models (55, 56). With zero-label language learning, no human-annotated data are used anywhere during training: the NLP models are trained purely on synthetic data generated from pretrained language models. For example, the “Unsupervised Data Generation” technique (56) uses a few user-supplied unlabeled examples to train a language model to synthesize high-quality training data without real human annotations; the model produces comparable results to baseline models trained on human-labeled data for classification problems. Furthermore, with the successes of foundational language models such as GPT-3, GPT-4 (57), and LLAMA (35, 58), there have been attempts to apply large language models directly to unlabeled data annotation task via prompt engineering and in-context learning; for example, (4) evaluates the capabilities of GPT-3 for different data annotation tasks such as classification and NER.

Weakly supervised learning approaches that depend on programmable rules and heuristics (i.e. labeling functions) to generate labels or synthesize new examples have also been explored (59); however, the technique works best on classification problems, and defining sensible rules is difficult.

The aforementioned approaches allow us to generate labeled training data for supervised and unsupervised ML training and validation; however, they either require some labeled examples to fine-tune the language models (4), work only for text classification, do not label the existing data directly, require significant postprocessing (56), or do not take into account the domain-specific nature of scientific texts, thus limiting their applicability to the labeling of domain-specific scientific artifacts for keyphrase extraction. Using pretrained language models for text labeling is also computationally intensive and often fails to cover the full diversity and complexity of real examples (47). Previous research has shown that large language models like GPT-3 may not perform well when applied directly to complex data annotation tasks such as NER without additional fine-tuning (4, 60), although properly contextualizing generative models with domain-specific knowledge is a promising approach for science tasks (at the risk of losing generalizability). Generative models also suffer from hallucinations (61), which make them less attractive for science tasks where reliability and reproducibility are critical.

The methods proposed in this work address some of these challenges. Our approaches generate labels for the existing training data rather than creating synthetic training data; they require no pretrained language models or labeled examples. A unique feature of the methods we present in this work is that our proposed techniques do not use ML techniques for the label generation/assignment task at all; instead, we primarily exploit real available public (human) knowledge such as related scientific works and controlled vocabularies. This has several advantages. First, our proposed approaches are particularly suited to handle and exploit the domain-specific nature of scientific texts, a task that would be more difficult with out-of-the-box pretrained general language models. Second, in addition to being computationally inexpensive, the methods developed here allow us to carry out keyword assignment and extraction tasks, not just text classification.

## Conclusion

In this work, we presented two approaches to automatically generate labels for validating ML-generated keywords for unlabeled texts. The first approach overcomes the lack of user-defined labels by exploiting direct links between scientific proposals and publications. In the second approach, we take advantage of domain-specific ontologies and frequency-based techniques to produce a set of derived labels against which the ML-generated keywords were validated. The results show varying degrees of success for both approaches based on the exact matching technique, with up to 44% of the link-derived keywords found by the ML algorithm and more than one in four of the extracted ML keyphrases found to be relevant. The approaches presented in this work can be applied to enhance data curation and improve the search of unlabeled texts in scientific databases and other information retrieval systems.

In the future, we plan to develop additional intelligent techniques for ranking the ontology-derived labels that incorporate semantic and contextual information. Furthermore, we plan to expand these approaches for validating unlabeled texts to other scientific artifacts (reports and theses) and other domains such as earth sciences.

## Appendix A: Generative model prompt for label generation

For our comparison against state-of-the-art generative models, we generate labels for the given input text using Vicuna-7B and LLAMA-13B. The prompt, in both cases, was to generate the best 10 keywords for the specified text.

For example, below is the exact prompt used for LLAMA-2:
